# Successful closure of thoracogastric airway fistula with occluder devices and coils under fluoroscopic guidance

**DOI:** 10.1055/a-2155-3369

**Published:** 2023-09-27

**Authors:** Yifan Li, Zongming Li, Yahua Li, Zhen Li, Kewei Ren

**Affiliations:** Department of Interventional Radiology, The First Affiliated Hospital of Zhengzhou University, Zhengzhou, Henan, China

The application of esophageal stent closure of fistulas is limited in application to thoracogastric airway fistulas due to changes in anatomical structure after surgery. Additionally, the unique location of three-branch orificium fistulas and large orificium fistulas can cause complications such as stent displacement and lax sealing 1 2 .

The patient in this case, a 56-year-old man, experienced a thoracogastric airway fistula after radical esophagectomy. Despite receiving prior esophageal stent treatment, the patient experienced a recurrence of symptoms 5 months post-surgery, worsening his nutritional status and quality of life. Given the patientʼs large fistula and the potential for incomplete sealing with airway stenting, a therapeutic regimen involving coils combined with occlusive devices was proposed.

After determining the location of the fistula ( Fig. 1 , Fig. 2 , Fig. 3 , Video 1 ), occlusion was performed using a 5-Fr vertebral catheter and hydrophilic film guidewire introduced through the fistula on the digestive tract side. A stiff guidewire was introduced and sent through the 8-Fr sheath tube to facilitate the introduction of a 9-mm septal occluder, which was accurately positioned and released. Further, the vertebral artery catheter was introduced into the right trachea through the gastropleural and tracheal fistula, and the delivery sheath was exchanged. Sequentially, the 6-mm ventricular septal occlusion device was employed to occlude the bronchothoracic fistula, followed by the 4-mm patent ductus arteriosus occlusion device to obstruct the fistula. Ultimately, the 8-mm patent ductus arteriosus occlusive device sealed the fistula. Additionally, three 6-mm × 14-mm coils were utilized to fill the gap of the occlusion device.

**Fig. 1:**
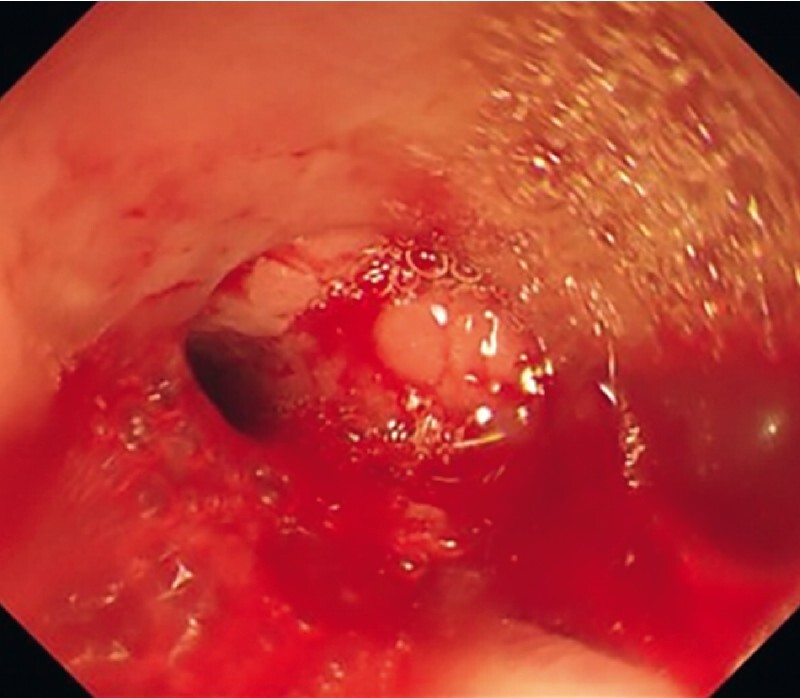
The fistula located at the superior carina was observed via bronchoscopy.

**Fig. 2:**
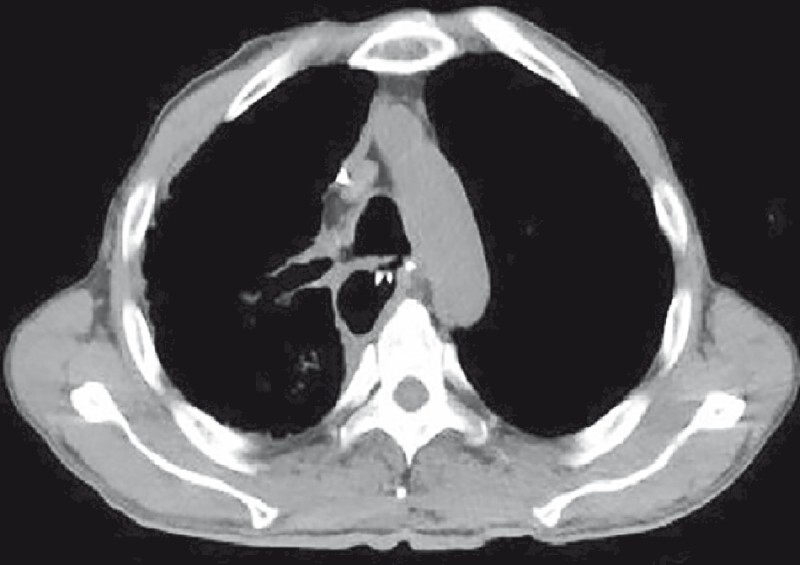
Chest computed tomography indicated that the bronchus exhibits communication with both the thorax and stomach.

**Fig. 3:**
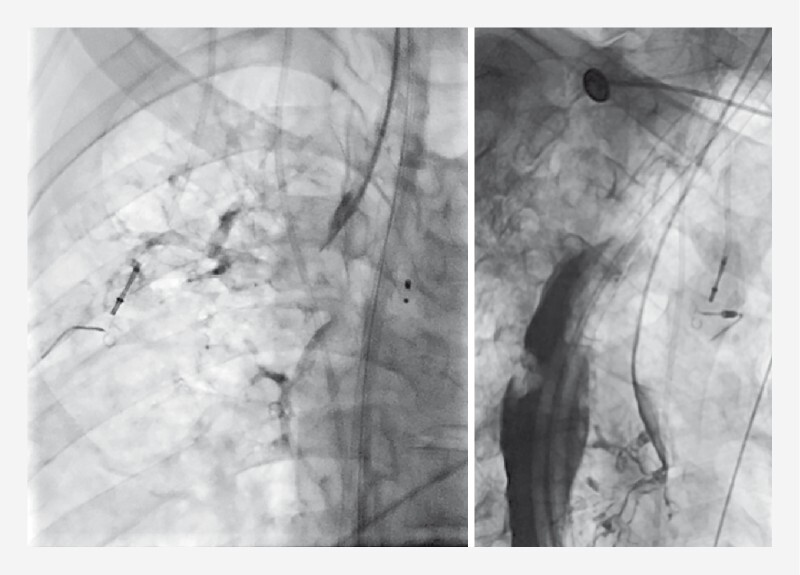
Following the administration of selective esophagography, the development of a bronchial contour was observed.

**Video 1** Thoracogastric airway fistula closed by occluder devices and coils under fluoroscopic guidance.


Re-examination demonstrated the successful closure of the thoracogastrotracheal fistula ( Fig. 4 ). The device offers effective physical obstruction for gas and gastric juice isolation, endothelial cell proliferation and granulation tissue adhesion. Compared to the airway stent, it exhibits reduced airway mucosal and ciliary damage, thereby mitigating the complications arising from sputum retention 3 4 .

**Fig. 4:**
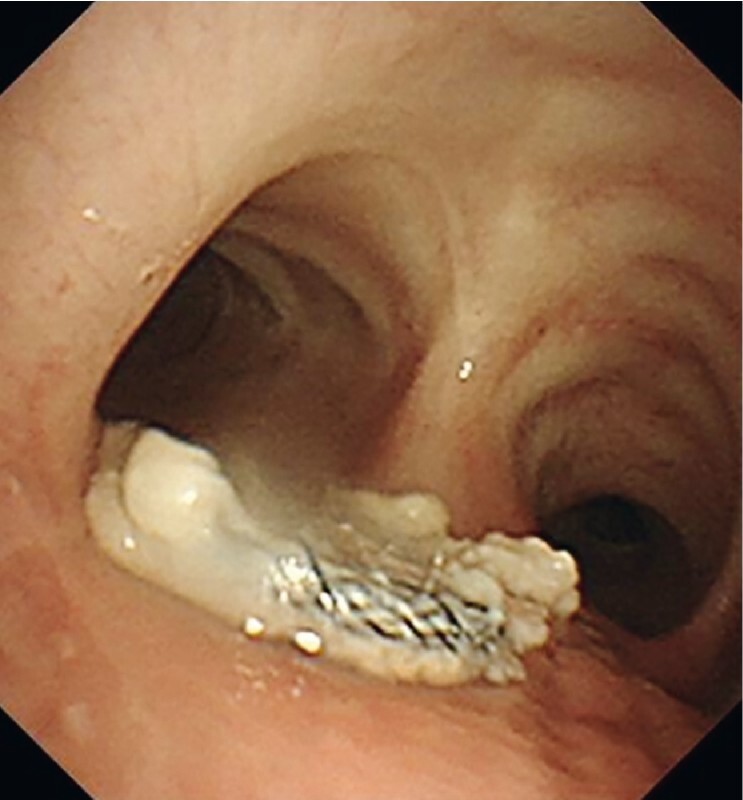
After a period of 6 months, bronchoscopy revealed the presence of the occluder device at the distal end of the trachea, with yellow sputum adhering to its surface and little granulation hyperplasia.

Endoscopy_UCTN_Code_CPL_1AH_2AG

## Notes

Endoscopy E-Videos

https://eref.thieme.de/e-videos
*E-Videos* is an open access online section of the journal *Endoscopy* , reporting on interesting cases and new techniques in gastroenterological endoscopy. All papers include a high-quality video and are published with a Creative Commons CC-BY license. Endoscopy E-Videos qualify for HINARI discounts and waivers and eligibility is automatically checked during the submission process. We grant 100% waivers to articles whose corresponding authors are based in Group A countries and 50% waivers to those who are based in Group B countries as classified by Research4Life (see: https://www.research4life.org/access/eligibility/ ). This section has its own submission website at https://mc.manuscriptcentral.com/e-videos
